# Supplementary material for: Mapping Physiological Suitability Limits for Malaria in Africa Under Climate Change
Source: Vector Borne Zoonotic Dis. 2015 Dec 1;15(12):718–25. doi: 10.1089/vbz.2015.1822 (PMC4700390; doi:10.1089/vbz.2015.1822)
Supplement: Supplemental data [file Supp_Fig1.pdf]

Supplementary Data

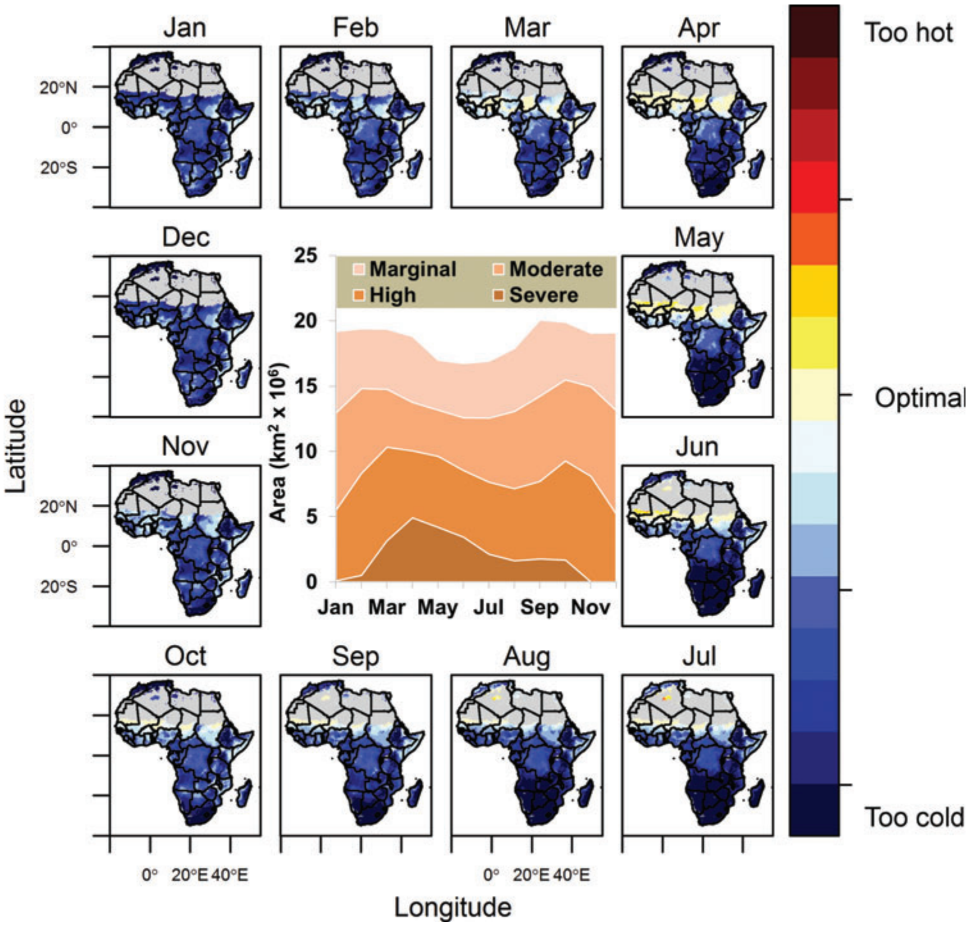

FIG. S1. Monthly temperature suitability for malaria transmission, as predicted by the thermal responses in Parham and Michael (2010). Temperature suitability is shown under the current climate (see Materials and Methods), on a blue–red scale from too cold for transmission (blue), through peak optimal transmission (white), to too hot for transmission (red). The aridity mask, where the area is unsuitable for mosquito development, is shown in grey, centered around the Sahara desert. (*Inset*) The area of land (in $km^2 \times 10^6$) in each month, within each quantile of transmission suitability, as described in the text.
