# Supplementary material for: Mapping Physiological Suitability Limits for Malaria in Africa Under Climate Change
Source: Vector Borne Zoonotic Dis. 2015 Dec 1;15(12):718–25. doi: 10.1089/vbz.2015.1822 (PMC4700390; doi:10.1089/vbz.2015.1822)
Supplement: Supplemental data [file Supp_Fig2.pdf]

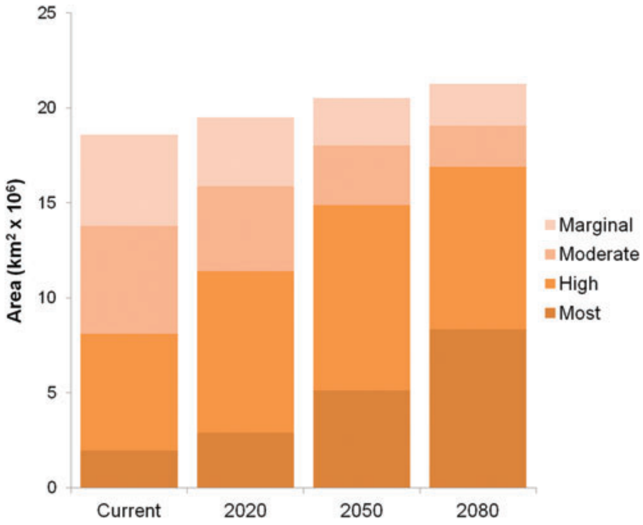

FIG. S2. The area of land on the African continent with temperatures within each transmission suitability quantile based on the thermal responses in Parham and Michael (2010). Transmissibility area quantiles averaged across months are shown as predicted by the thermal responses for current and future (SRES A1B) climate scenarios—2020, 2050, and 2080—as described in Materials and Methods.
